# Rapid Improvement Following Receipt of Infliximab in Steroid-refractory Durvalumab-Associated Grade 3 Pneumonitis

**DOI:** 10.7759/cureus.22295

**Published:** 2022-02-16

**Authors:** Sherri Huang, Aryanna Jordan, Dakota Jenneman, Michael Shafique, Bjorn Holmstrom

**Affiliations:** 1 Internal Medicine and Pediatrics, University of South Florida Morsani College of Medicine, Tampa, USA; 2 Internal Medicine, University of South Florida Morsani College of Medicine, Tampa, USA; 3 Oncology, Levine Cancer Institute, Carolinas Medical Center, Tampa, USA; 4 Medical Oncology, Moffitt Cancer Center, Tampa, USA; 5 Internal Medicine, Moffitt Cancer Center, Tampa, USA

**Keywords:** infliximab, drug induced pneumonitis, immune-related adverse event (irae), cancer immunotherapy, non-small cell lung carcinoma (nsclc)

## Abstract

Immune checkpoint inhibitors (ICIs) are important novel agents used in advanced non-small cell lung cancer (NSCLC) standard regimens; however, their use increases the risk of immune-related adverse effects (IRAEs). The incidence of IRAE pneumonitis is well documented in ICI use. Corticosteroids continue to be the mainstay of treatment for IRAEs. Here we report one of the first cases of using infliximab to treat durvalumab-associated pneumonitis.

## Introduction

In the last decade, the immunotherapy agents nivolumab, pembrolizumab, and atezolizumab became the first immune checkpoint inhibitors (ICIs) to be added to advanced non-small cell lung cancer (NSCLC) standard regimen, which previously centered on chemoradiation with or without surgical resection. These immunotherapy agents block the interaction between the PD-1 ligand (PDL-1) and PD-1 receptor by inhibiting either PD-1 (nivolumab and pembrolizumab) or PDL-1 (atezolizumab and durvalumab), and this inhibition, in turn, promotes the ability of T-cells to recognize and destroy tumor cells [1]. Durvalumab received FDA approval in 2018 as consolidation therapy in stage 3 disease after chemoradiation in tumor cells with >1% PDL-1 expression [2,3].

Immune-related adverse events (IRAEs) are a unique spectrum of side effects of ICIs, which resemble autoimmune responses that can affect almost every organ of the body and include dermatologic (rash), gastrointestinal (diarrhea and colitis, hepatitis and pancreatitis), endocrine (thyroiditis and diabetes), pulmonary (pneumonitis), musculoskeletal (myositis and arthritis) and other inflammatory manifestations [4,5]. The grading system for IRAEs is categorized by clinical severity and assigned from grade 1 to grade 5. For example, grade 1 pneumonitis is defined as asymptomatic, clinical, or diagnostic observations only with no interventions indicated; and grade 5 is defined as death. Grade 3 is defined as severe symptoms which limit self-care activities of daily living and oxygen is indicated. In a meta-analysis, PDL-1 inhibitor incidence of any grade pneumonitis was found to be 1.3% [6]. In a meta-analysis that evaluated the types of IRAEs that were fatal, the incidence of fatal pneumonitis associated with anti-PDL-1/anti-PD-1 monotherapy was 35% [7].

The association between durvalumab and pneumonitis is well-documented. The phase 3 PACIFIC trial compared patients who received consolidation durvalumab versus placebo after chemoradiotherapy for stage III unresectable NSCLC, finding that the incidence of any grade of pneumonitis or radiation pneumonitis was 33.9%. In this study, the incidence of grade 3 or 4 pneumonitis was 3.4% and 2.6%, respectively [8]. The rate of grade 2 or above radiation pneumonitis after post-concurrent chemoradiotherapy durvalumab was 18% in one study and 36% in another [9,10]. Finally, pneumonitis was the most common grade 3 or 4 IRAE (1%) in a prospective study of 444 patients who received durvalumab [11]. 

The clinical course of IRAE management varies across the literature. Infliximab is recommended for treating steroid-refractory pneumonitis [12]. The mechanism of action of infliximab is an inhibitor of TNF-alpha, which has been posited to be involved in the local injury and inflammatory response in inflammatory lung diseases and therefore may represent a possible mechanism of action in immune-mediated pneumonitis [13]. Here we present a case of a patient who experienced rapid improvement following receipt of infliximab for grade 3 durvalumab-associated pneumonitis.

## Case presentation

The patient is a 68-year-old with a history of stage 3 NSCLC, specifically left upper lobe pulmonary carcinoma who presented with worsening cough and dyspnea of a few months' duration after initiation on durvalumab. He is a former 90-pack-year smoker. His other medical history included chronic obstructive pulmonary disease (COPD), coronary artery disease, carotid artery stenosis, hypertension, and diabetes mellitus. 

**Figure 1:**
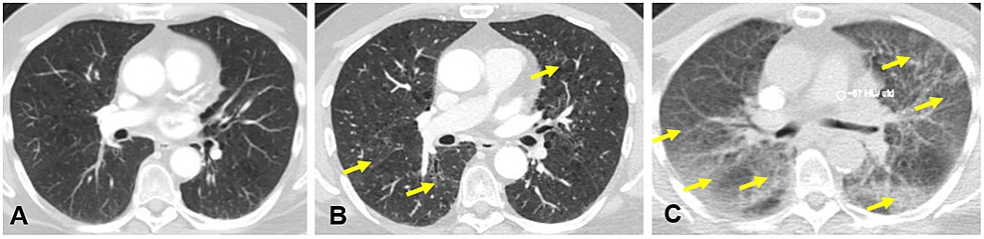
Comparison of computerized tomography (CT) thorax images before and after initiation of consolidation therapy (A) CT thorax with contrast 10 days prior to durvalumab initiation. (B) CT thorax with contrast showing new opacities (arrows) following the patient’s development of dyspnea. (C) CT angiogram on admission for this case report, showing worsened and new diffuse opacities (arrows) following antibiotic and steroids course, prior to infliximab.

Prior to treatment, the lung lesion measured about 1.6 x 3.4 x 1.6 cm with a PET scan showing avidity also in an anterior left hilar region lymph node; no avidity was noted elsewhere to suggest metastatic disease. The patient underwent concurrent chemoradiation with two cycles of cisplatin and pemetrexed and radiation at a total dose of 70 Gray in 35 fractions. Following this, he received two cycles of consolidation durvalumab. After two cycles of durvalumab, treatment was held as he experienced increased cough and shortness of breath. He tested negative for COVID-19, and a CT scan demonstrated new consolidative ground-glass opacities in the right lung suspicious for pneumonitis of infectious versus inflammatory etiology (Figure 1A and Figure 1B). He was treated with levofloxacin with an improvement of symptoms; however, his cough and dyspnea worsened after this period of partial improvement. He was subsequently placed on amoxicillin/clavulanic acid and a prednisone taper. Continuing symptoms precluded the resumption of durvalumab. After obtaining the second dose of the COVID-19 vaccine and experiencing chills, rigors, and severe headaches, the patient paused all his medications. He presented in the urgent care center with oxygen saturation in the 80% range on room air which improved to a low 90% range on 3 liters oxygen supplementation. He was afebrile, hemodynamically stable; labs showed no leukocytosis and procalcitonin within normal limits. A respiratory virus panel that included COVID-19 was negative. CT angiogram now showed new bilateral diffuse consolidative and ground-glass opacities (Figure 1C). 

The patient received a day of 60 mg IV methylprednisolone twice daily with little symptomatic or clinical improvement. IV methylprednisolone was subsequently increased to 80 mg twice daily. In addition, after an informed discussion about the medication with the primary team, he received one dose of 420 mg (about 5 mg/kg) IV infliximab. The following day, he subsequently reported decreasing shortness of breath and cough, and two days later demonstrated a decrease in oxygen supplementation requirements to room air. He was discharged four days after admission. At discharge, given the high risk of recurrent pneumonitis, his durvalumab continued to be held. He was placed on surveillance imaging. About eight months after discharge, his surveillance CT showed an enlarging left upper lobe mass which was concerning for progressive disease versus post-therapy related changes, and he underwent interventional radiology-guided biopsy of the mass, the results of which are still pending as of this writing. Per available electronic health records at this time, he has not had repeated emergency room or hospitalizations for dyspnea and on the last follow-up remained on room air.

## Discussion

Herein, we present a 68-year-old with a history of stage 3 pulmonary carcinoma who presented with likely inflammatory etiology of his worsening dyspnea that began after two cycles of consolidation therapy with durvalumab. To our knowledge, our patient represents the first report of infliximab use in durvalumab-associated pneumonitis and supports the useful role of infliximab in IRAE pneumonitis. Prior to this case report, infliximab has been used successfully in the context of durvalumab-associated hepatitis and in pembrolizumab-induced pneumonitis [14,15]. Moreover, although infliximab ameliorated post-nivolumab pneumonitis in a head and neck cancer patient, our report is unique given our patient had pre-existing pulmonary pathology [16]. COPD and history of radiotherapy, as in this patient, are risk factors for IRAE pneumonitis [17]. Nevertheless, there is limited literature on the efficacy of infliximab across IRAE pneumonitis patients with various pre-existing pulmonary conditions. Our case provides one example of the successful use of infliximab in an IRAE pneumonitis patient with pre-existing COPD and a history of chemoradiation for his NSCLC. 

Furthermore, the rapid improvement after a single infliximab dose in this grade 3 pneumonitis NSCLC patient who had adhered to a partial outpatient prednisone taper and an inpatient IV methylprednisolone course is noteworthy. In a retrospective study with patients who underwent locally advanced NSCLC followed by durvalumab, all patients with grade 1 or 2 pneumonitis who received 0.5 mg/kg of oral corticosteroids experienced clinical improvement. However, the same study included one grade 3 pneumonitis patient, who passed away despite receiving 1 mg/kg steroid therapy [18]. In a study of 65 patients with pneumonitis after ICI for various cancers, it was found that 18.5% experienced a steroid-refractory course after 48 hours of high-dose corticosteroids. Those patients were treated with IVIg, infliximab, or a combination of IVIg and infliximab. Although the study did not provide information on the immunotherapy agents associated with the ICIs, 53% of patients treated with infliximab alone required mechanical ventilation, and all five patients treated with infliximab died from their ICI-pneumonitis or infectious complications [19]. In another study evaluating 2,750 patients with lung cancers experiencing IRAE, improvements on steroids and additional immunosuppressants were less common for patients who developed pneumonitis and neuromuscular inflammation compared to patients who developed hepatitis and colitis [20]. Together, these observations demonstrate that the clinical course during the management of IRAEs varies and may depend on IRAE type and grade.

## Conclusions

This case presentation demonstrates the rapid clinical improvement in a patient administered infliximab to treat steroids-refractory pneumonitis. Based on this case and prior literature, further study and stratification of patient characteristics including response to steroids response is needed to provide a prognosis of IRAE management. Our case report suggests that depending on the IRAE type and grade, infliximab may represent a promising intervention for steroids-refractory grade 3 IRAE pneumonitis.
